# Expression Localization of the *KRT32* Gene and Its Association of Genetic Variation with Wool Traits

**DOI:** 10.3390/cimb46040185

**Published:** 2024-03-30

**Authors:** Zhanzhao Chen, Fangfang Zhao, Zhaohua He, Hongxian Sun, Qiming Xi, Xueqin Yu, Yuan Ding, Ze An, Jiqing Wang, Xiu Liu, Mingna Li, Zhiyun Hao, Shaobin Li

**Affiliations:** Gansu Key Laboratory of Herbivorous Animal Biotechnology, Faculty of Animal Science and Technology, Gansu Agricultural University, Lanzhou 730070, China; chenzc@st.gsau.edu.cn (Z.C.); zhaofangfang@gsau.edu.cn (F.Z.); hezh@st.gsau.edu.cn (Z.H.); sunhx@st.gsau.edu.cn (H.S.); xqm5217463@163.com (Q.X.); yuxq@st.gsau.edu.cn (X.Y.); dingy@st.gsau.edu.cn (Y.D.); anz@st.gsau.edu.cn (Z.A.); wangjq@gsau.edu.cn (J.W.); liuxiu@gsau.edu.cn (X.L.); limn@gsau.edu.cn (M.L.); haozy@gsau.edu.cn (Z.H.)

**Keywords:** Gansu Alpine fine-wool sheep, keratin, wool traits, SNP, immunofluorescence

## Abstract

Changes in keratin gene expression and spatiotemporal regulation determine the compositional content and cellular localization of wool keratin, thereby affecting wool traits. Therefore, keratin gene family member 32 (*KRT32*) was selected for a study using RT-qPCR, immunofluorescence, and penta-primer amplification refractory mutation system (PARMS) techniques. The results showed that *KRT32* mRNA was highly expressed in the skin and localized to the inner root sheath (IRS), outer root sheath (ORS) and dermal papilla (DP). Sequencing results identified eight SNPs in *KRT32*, and association analyses revealed that the variations were significantly associated with multiple traits in wool (*p* < 0.05), including MFD, CF and MFC. The constructed haplotype combination *H_2_H_3_* has higher CF and smaller MFD than other haplotype combination (*p* < 0.05). In conclusion, *KRT32* can be used as a candidate gene for molecular genetic improvement of wool in Gansu Alpine Fine-wool sheep.

## 1. Introduction

Wool is one of the most moisture-sensitive natural fibers [1] and is unique in nature in its ability to both absorb moisture and wick away perspiration. According to X-ray diffraction analysis, the structure of keratin consists of an α-helical structure and a β-folded lamellar structure [2]. There are two main types of wool keratin: one is the alpha-helix-based keratin intermediate filament proteins (IFPs), which act as the wool backbone and account for 58% of the total wool proteins; the other is keratin-associated proteins (KAPs), which serve as matrix components. These two proteins are cross-linked [3]. Keratin is particularly chemically stable and possesses high mechanical strength [4] because it contains a high level of cystine and a particularly high level of disulfide bonds, which act as cross-links in the peptide chain of the protein. The physical properties of wool fibers are a very important factor in the grading and classification of wool for different textile applications [5]. Wool diameter is a key factor in determining the quality and value of wool, and almost all properties of wool are related to or directly determined by fineness, such as strength, elongation, elasticity, bending stiffness and moisture absorption among other properties. Wool is a naturally curly animal fiber and its curvature is an important criterion for assessing the quality and craft value of wool. The natural curvature of wool’s fibers defines it from other fibers. It is precisely because of its natural curl that wool can form many intervals of immobile space as a barrier to warmth. Therefore, in order to increase the economic value of wool, it is important to improve wool traits. 

Keratins (*KRTs*) and Keratin-associated proteins (*KRTAPs*) are the most strongly expressed gene families in the hair follicle bulb [6]. During hair follicle development, *IFPs* are expressed first, followed by the *KRTAPs*, and the total protein expressed by the two gene families undergoes a complex process of keratinization to form wool. The amount of IFPs in different qualities of wool is relatively stable and it is difficult to distinguish differences in IFPs from differences in the protein fractions of different wools [7]; therefore, there are fewer studies based on the relationship between IFP protein differences and gene expression. Although there are many keratin genes that regulate the same wool trait [8], they are different at the DNA level. Changes in keratin gene expression and spatio-temporal regulation determine the compositional content and cellular localization of wool keratin, thereby influencing traits such as curvature, fiber diameter, strength and length of wool. Related studies have suggested that variation in wool traits is based on changes in genes and the proteins they encode, and that changes in genes lead to phenotypic differences in wool [9]. *KRTs* play an essential role in follicle development and in the formation of quality traits in wool [10], and most their variations correlate with wool structure and fiber properties, making them ideal candidates for improving wool traits. In this study, a penta-primer amplification refractory system (PARMS) was used for sheep genotyping to investigate the effect of keratin gene variation on wool traits in Gansu Alpine Fine-wool sheep. Genetic polymorphisms of the *KRT32* (exons 1, 6 and 7) were analyzed and genotypes and haplotype combinations were analyzed for association with wool traits. The results may provide a theoretical basis for improving wool quality.

## 2. Materials and Methods

### 2.1. Collection of Wool, Blood and Tissue Samples from Sheep Samples

Two hundred and forty-two Gansu Alpine Fine-wool sheep ewe lambs from the same growing environment were selected for genetic variation for the *KRT32*. Blood was collected from the neck of the sheep and a portion of it was loaded onto an FTA card for DNA extraction using a two-step process [11]. At the first shearing (12 months of age), wool samples were collected from the sheep at the position of the posterior margin of the left scapula and wool traits were measured by New Zealand Pastoral Measurements LTD (Ahuriri, Napier, New Zealand), including mean fiber diameter (MFD), fiber diameter standard deviation (FDSD), coefficient of variation of fiber diameter (CVFD); mean staple length (MSL), mean fiber curvature (MFC), mean staple strength (MSS) and comfort factor (CF).

Three newborn Gansu Alpine Fine-wool sheep ewes were selected and skin samples were collected from the posterior margin area of the shoulder joint of the left front leg on days 1, 30, 60, 90, 180, and 270 using a skin sampler with a radius of 0.44 cm; one part was placed in a freezing tube containing 4% paraformaldehyde and stored into a 4 °C ice box for immunofluorescence analysis, and the other part was placed in a freezing tube containing RNA protection solution for subsequent RT-qPCR analysis. In addition, three adult ewes in good physical condition were selected, tissue samples were taken and placed in cryopreservation tubes containing RNA-protecting solution for subsequent RT-qPCR analysis. 

### 2.2. RT-qPCR Analysis

Total RNA was extracted from the collected tissues using Trizol reagent (Shanghai Yuanye Bio-Technology Co., Ltd., Shanghai, China) and cDNA was obtained by reverse transcription using an EvoM-MLV Reverse Transcription Kit (Accurate Biology, Wuhan, China), −20 °C ambient storage. The Beta-actin(ACTB) gene was used as a reference to calibrate the level of gene expression, and primers for the target gene were designed using Primer software (Primer Premier 5.0, Prime Corporation, Vancouver, BC, Canada) based on the gene data published in NCBI, and the primer sequences and PCR conditions are shown in Table 1. We analyzed the concentration, purity, and integrity of the RNA before doing RT-qPCR to ensure that the quality of the RNA supports the subsequent results.

### 2.3. Immunofluorescence Analysis

The primary antibody used in the experiment was K32 (TD9003, rabbit antibody) and the secondary antibody was CY3 (GB21303, goat anti-rabbit IgG); primary antibody was provided by Abmart PharmaTech Ltd. (Shanghai, China) and secondary antibody was provided by Servicebio Technology Co., Ltd. (Wuhan, China). Skin samples in good basic structural condition were selected, trimmed, dehydrated, paraffin-embedded, and sectioned. Paraffin sections were dewaxed using gradient biodewax and clear solution, anhydrous ethanol and distilled water. Antigen repair was performed using ethylene diamine tetra-acetic (EDTA) acid antigen retrieval buffer antigen repair solution (pH = 8). The slides were placed in PBS (pH 7.4) on a decolorizing shaker and shaken and washed 3 times for 5 min each. Sections were slightly shaken dry and circled around the tissue with a histochemical pen and sealed with a drop of 3% Bovine Serum Albumin (BSA) for 30 min. The primary antibody was added dropwise and the sections were incubated flat in a humid box at 4 °C overnight. The slides were washed in PBS (pH = 7.4) on a decolorizing shaker with shaking for 3 times, each time for 5 min, then the corresponding secondary antibody was added and incubated for 50 min at room temperature, protected from light. The slide wash was repeated under the same conditions and then the DAPI stain was dropped in and incubated for 10 min away from light. Then, we washed it again and added the tissue autofluorescence quench B solution for 5 min and rinsed with running water for 10 min. Sections were dried and sealed with an anti-fade sealant, and images were collected and analyzed.

### 2.4. PCR Amplification and Genotyping

According to the Ensembl database (https://www.ensembl.org (accessed on 2 March 2024)), polymorphism prediction showed that the polymorphisms of *KRT32* gene were mainly concentrated in exons 1, 6 and 7, so these three exon fragments were selected as the analysis fragments. Primers for three exons were designed using the Primer software (Table 2). A total of 20 genomic DNAs of Gansu Alpine Fine-wool sheep were used to amplify three exons and all amplicons were then sequenced. The Primer synthesis, amplification, sequencing, and genotyping were performed by Gentides Biotech Co., Ltd. (Wuhan, China). Genotyping was performed using a penta-primer amplification refractory system (PARMS), a SNP PCR analysis technique that combines a pair of universal fluorescent primers, a pair of SNP allele-specific primers, and a reverse shared primer. Different primers (Table 3) bind specifically to FAM and HEX fluorescent markers in the PARMs master mix, respectively, and then fluorescence scanning is performed at the end of the reaction and the presence of alleles was determined based on the corresponding fluorescence signals. 

### 2.5. Statistics and Analysis

After successful genotyping of *KRT32*, allele frequency, genotype frequency and polymorphism information content (PIC) were calculated using the formula described by Botstein et al. [12]. Haploview 4.2 was used for linkage disequilibrium analysis and haplotype construction, after eliminating the results from sheep that could not be typed. One-way analysis of variance (ANOVA) was used to reveal the association between the frequency of Gansu Alpine Fine-wool sheep genotypes and wool traits. The results were output as mean ± standard error (S.E.), with *p* < 0.05 as the criteria for determining significant differences. The RT-qPCR results were calculated in excel using the 2^−ΔΔCT^ method.

## 3. Results

### 3.1. Expression of KRT32 mRNA in the Skin of Different Tissues and Stages of Gansu Alpine Fine-Wool Sheep

**Figure 1:**
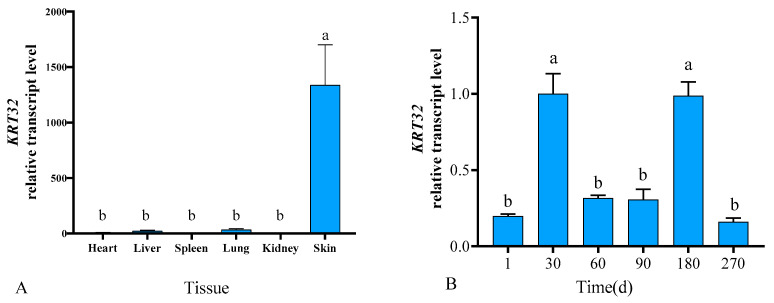
(**A**,**B**) indicate the expression results of *KRT32* mRNA in different tissues and different skin stages in Gansu Alpine Fine-wool sheep, respectively. The data are expressed as the mean ± S.E. Different letters indicate significant differences between different ages (*p* < 0.05).

The OD260/OD280 of RNA were all between 1.8 and 2.0 and the concentrations were all greater than 100 ng/μL, which indicating that the RNA could be used for subsequent experiments. RT-qPCR results showed that *KRT32* was expressed in heart, liver, spleen, lungs, kidneys and skin, and mRNA levels were significantly higher in skin tissues than in other tissues (*p* < 0.01). The *KRT32* is expressed in the skin at all six periods of time, with expression increasing at birth, decreasing and stabilizing on days 30–90, increasing again on days 90-180, and decreasing again after day 180. In general, the expression was alternately increasing or decreasing. (*p* < 0.05; Figure 1).

### 3.2. Expression Localization and Distribution Density of KRT32 Encoding Protein in SKIN Tissues of Gansu Alpine Fine-Wool Sheep of Various Ages

**Figure 2:**
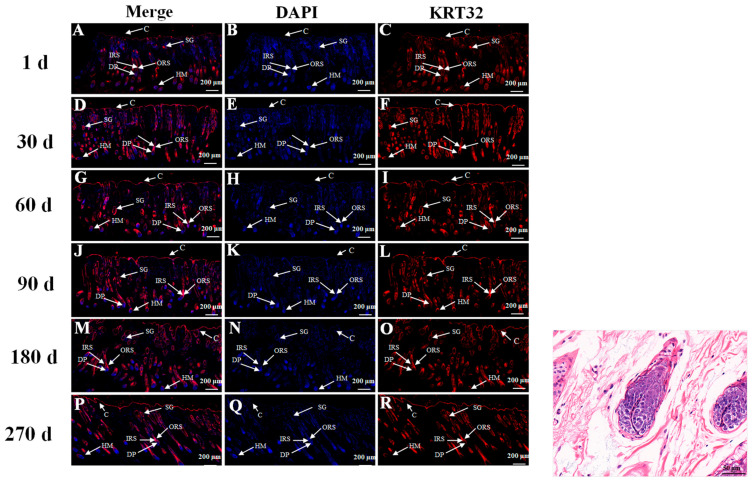
Immunofluorescence staining for K32 in skin tissues of Gansu Alpine Fine-wool sheep at different times (5×). Blue (**B**,**E**,**H**,**K**,**N**,**Q**) and red (**C**,**F**,**I**,**L**,**O**,**R**) tissue in the figure show DAPI-labeled nuclear fluorescence staining and fluorescence staining for K32, respectively. ORS: outer root sheath; IRS: inner root sheath; HM: hair matrix; C: corneum; SG: sebaceous glands; DP: dermal papilla. H&E staining image are shown on the right. The first column (**A**,**D**,**G**,**J**,**M**,**P**) is a combined graph of the second and third columns.

The expression and distribution of the *KRT32*-encoding protein was analyzed using immunofluorescence combined with the positive scoring of different parts of the hair follicle. Distribution of *K32* in skin tissues of Gansu Alpine Fine-wool sheep of different ages was analyzed using immunofluorescence. The image on the right shows the H&E staining of the skin histology of Gansu alpine fine wool sheep to facilitate visualization of the entire hair follicle structure (Figure 2). The results showed that the cuticle, IRS, ORS, HM and DP were strongly positively expressed in the skin tissues of Gansu Alpine Fine-wool sheep at 1 day old. At 30, 60, 90, 180 and 270 days of age, skin tissue were high positive expression in the IRS, ORS, HM, sebaceous glands and DP (Table 4).

### 3.3. Genotyping and Polymorphism Analysis 

**Figure 3:**
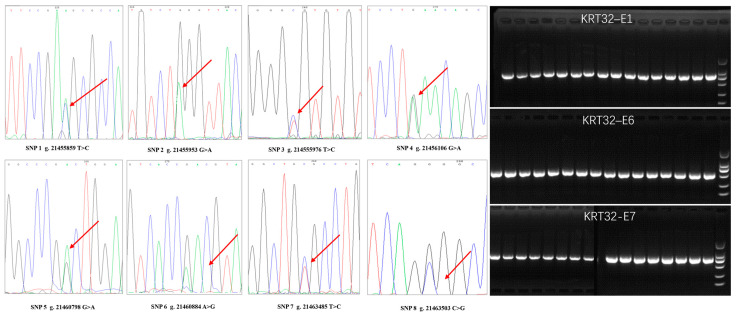
PCR amplification of exons 1, 6 and 7 of *KRT32* and sequencing results, overlapping peaks indicate SNPs. The overlapping peaks in the direction of the arrows refer to mutation sites, and the different colored lines represent different bases.

The *KRT32* exons 1, 6 and 7 were sequenced in 20 randomly selected DNA samples of Gansu Alpine Fine-wool sheep. Eight SNPs were detected: SNP1 (g.21455859), SNP2 (g.21455953), SNP3 (g.21455976), SNP4 (g.21456106), SNP5 (g.21460798), SNP6 (g.21460884), SNP7 (g.21463485) and SNP8 (g.21463503) (Figure 3). Of the 8 SNPs, three of them located in non-coding regions (SNP1, SNP7, SNP8) and five in coding regions (SNP2, SNP3, SNP4, SNP5 SNP6), of which three were synonymous (SNP2, SNP4, SNP6) and two were missense mutations (SNP3, SNP5). The GTG codon to GCG codon change in SNP3 results in the change of valine to alanine; hence, p.Val17Ala, and the GAC codon to AAC codon change in SNP5 results in the change of aspartic acid to asparagine, defined as p.Asp17Asn. The length of the DNAmarker for electrophoresis was 2000 bp. The PCR analysis produced a fragment of 670 bp, 612 bp and 468 bp, respectively, which were of the expected size (Figure 3).

**Figure 4:**
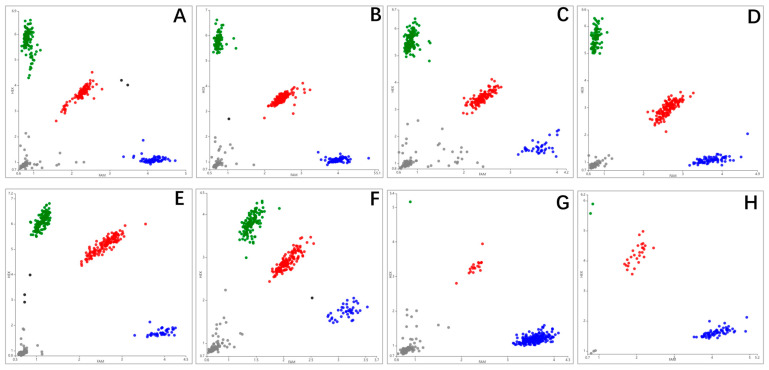
Genotyping results for eight positions of the *KRT32* (**A**–**H**) gene in Gansu Alpine Fine-wool sheep. Different colors represent different genotypes, with green and blue representing heterozygotes and red representing purities.

Genotyping using the PARMS technique revealed all the eight SNPs showed three genotypes; heterozygotes are located in the middle of the scatterplot and are indicated by red dots, while pure heterozygotes are located at the edges of the scatterplot, close to the x-axis and y-axis, and are indicated by blue and green dots, respectively. Grey dots are generally wells with no DNA samples or wells with no signal for PCR amplification (Figure 4). The horizontal coordinate is the FAM fluorescence value, and the vertical coordinate is the HEX fluorescence value. The dominant alleles of the eight SNPs in the *KRT32* were C, A, C, A, A, G, T and C, corresponding to the genotypes with the highest frequencies as CC (0.402), GA (0.480), TC (0.452), GA (0.480), GA (0.452), AG (0.402), TT (0.712), CC (0.887). Population genetic analysis of eight positions in Gansu Alpine Fine-wool sheep revealed that all positions were moderately polymorphic (0.25 < PIC < 0.5), except SNP8 which was lowly polymorphic (PIC < 0.25) (Table 5).

### 3.4. Analysis of the Association of SNPs with Wool Traits

Eight SNPs for the *KRT32* was analyzed for association with wool traits (Table 6). SNP1, SNP 3 and SNP 5 were significantly correlated with the coefficient of CVFD and MFC (*p* < 0.05); SNP 2, SNP 4 and SNP 6 were significantly correlated with MFC (*p* < 0.05); SNP7 was significantly correlated with the CVFD (*p* < 0.05); SNP8 was significantly associated with MFD and CF (*p* < 0.05). MFC was significantly higher in individuals with TT genotype than CC genotype on SNP1 (*p* < 0.05); MFC was significantly higher in individuals with GG genotype than AA genotype on SNP2 (*p* < 0.05); MFC was significantly higher in individuals with TT and TC genotypes than CC genotypes on SNP3 (*p* < 0.05); CVFD was significantly higher in individuals with a TC genotype than CC genotype, and other MFC was significantly higher in GG and GA genotypes than in AA genotypes on SNP4 (*p* < 0.05); MFC was significantly higher in individuals with GG and GA genotypes than in AA genotypes on SNP5, and CVFD was significantly higher in individuals with GA genotypes than in AA genotypes on SNP6 (*p* < 0.05); MFC was significantly higher in AG and AA genotypes than in GG genotypes on SNP6. In SNP7, the CVFD of TT genotypes was significantly higher than that of TC genotypes; in SNP8, the MFD of CG genotypes was significantly higher than that of GG genotypes, and the CF of CC genotypes was significantly higher than that of CG genotypes (*p* < 0.05).

### 3.5. Haplotype Reconstruction of Gene SNPs and Association Analysis of Haplotype Combinations with Wool Traits

**Figure 5:**
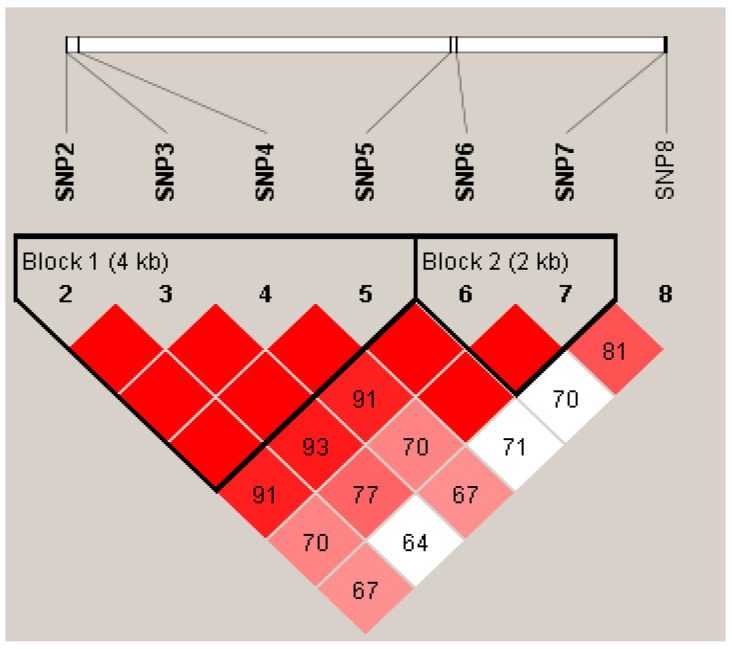
Linkage disequilibrium analysis of SNPs in *KRT32.* The color of the square indicates the degree of interlocking, and the darker the color, the higher the degree of interlocking; The value represents the strength (percentage) of the correlation value between SNPs.

Eight SNPs of the *KRT32* were screened and analyzed for linkage disequilibrium and haplotype using the Haploview 4.2 software. The results showed that among the eight SNPs in *KRT32*, SNP1 and SNP8 were not involved in haplotype formation due to their weak association with other mutation sites, and the remaining six SNPs were screened to form two haplotype blocks, that is, Block1 (g.21455953 G>A, g.214555976 T>C, g.21456106 G>A and g.21460798 G>A) and Block2 (g.21460884 A>G and g.21463485 T>C), respectively, and both of them were in the strong interlocking state (Figure 5). In the Gansu Alpine Fine-wool sheep population, four haplotypes were constructed in Block1, and these haplotypes were combined to form six haplotype combinations with frequencies greater than 0.01 (Table 7). Three haplotypes were constructed in Block2, and combining these haplotypes together similarly resulted in six haplotype combinations with frequencies greater than 0.01 (Table 8). 

Associations with wool traits were analyzed for each of the six haplotype combinations of Block 1. The results showed that different haplotype combinations of four SNPs (SNP2, 3, 4 and 5) of the *KRT32* were significantly correlated with MFC; different haplotype combinations of 2 SNPs (SNP6 and SNP7) of the *KRT32* were significantly correlated with MFD, MFC and CF. The MFC of *H_2_H_2_* and *H_2_H_3_* individuals in Block1 of the *KRT32* was significantly higher than that of *H_3_H_3_* individuals (Table 9); In Block2, MFD was significantly higher in *H_1_H_3_* individuals than in *H_2_H_3_* individuals, while the CF was significantly lower in *H_1_H_3_* individuals than in *H_2_H_3_* individuals, MFC was lower in *H_1_H_3_* individuals than in *H_1_H_2_*, *H_2_H_2_*, *H_2_H_3_* and *H_3_H_3_* individuals (Table 10).

## 4. Discussion

Studies on the use of polymerase chain reaction-single-stranded conformational polymorphism (PCR-SSCP) to identify the effect of SNPs in *KRT83* [13] and *KRT31* [14] on wool traits have demonstrated nucleotide sequence variation in the sheep keratin genes. Analysis of *KRT32* in Ensembl (version 110, 2023) showed that the gene is located on chromosome 11, and there is a sequence in the Rambouillet v1.0 genome construct CM008482.1 (a fine wool sheep breed), identified as ENSOARG00020008724 (GenBank GENE ID: 100526788), which is close to another keratin gene, *KRT35* (ENSOART00020008687). The ATG start site of the *KRT32* sequence is located in exon 1, with a predicted length of 401 amino acids.

Wool fibers consist mainly of keratin, encoded by the *KRT* and *KRTAP* genes, which are expressed in a highly regulated manner during hair follicle growth, as reflected by their ordered spatiotemporal distribution of mRNA expression. This study examined the expression levels of *KRT32* mRNA in different other organs and in skin at different periods using RT-qPCR. The results showed that the expression level of *KRT32* mRNA in skin was significantly higher than that in parenchyma organs, and it was expressed in skin at different developmental periods, with significant differences in the expression levels at various ages. It’s related to the hair cycle. The expression of *KRT32* increased to a peak during the anagen phase and then decreased to a steady state. During the catagen phase, the expression of *KRT32* increased to a peak and then decreased, therefore, so it was inferred that *KRT32* may regulate the maturation of hair follicles and wool growth mainly during the anagen and catagen phases.

Hair follicle growth is a complex cyclical physiological process influenced by a variety of factors. The feasibility of using fluorescent antibodies techniques to localise keratin in hair follicles was established as α-keratin has been shown to be antigenic [15,16,17]. In this study, immunofluorescence was used to determine the location of protein expression, and the results showed that *KRT32* mainly expressed in the DP, IRS, ORS and HM, with relatively weak expression in the cuticle, which is basically in line with the results of previous studies on the localization of mRNA expression [18]. Positive scores showed that the protein encoded by the *KRT32* is distributed at a relatively uniform density in all structures of the hair follicle except the stratum corneum, and since DP cells and ORS cells are the major cell types that regulate hair growth and regeneration [19], thus the KRT32 may have an important effect on follicle maturation and wool growth.

Chen et al. [20] elucidated that *KRT75* plays a critical role in maintaining the hair shaft by constructing a mouse model. Chai et al. [21] revealed that variation in the promoter fragment of *KRT34* affects MFD, FDSD, and MSL, and Bao et al. [10], in an experiment using yak as a test subject, discovered that *KRTs* showed a strong correlation throughout the hair follicle development cycle, suggesting a generalised effect of keratin on the hair growth process in different species. In this study it was found that KRT32 was significantly correlated with MFD, MFC, both of which are major traits determining the value of wool. We confirmed the genetic poly-morphism of the *KRT32* in sheep, and a total of eight SNPs were screened in the three exons. Three of the variants occurred in the non-coding region, including one in the 5′-UTR and two in the 3′-UTR; and other five variants were identified in the coding region, including three synonymous and two missense mutations. SNPs in the coding region may alter the amino acid, affecting the structure and function of the corresponding proteins [22], ultimately leading to changes in the wool phenotype. In this study, we found that SNP3 and SNP5 in the coding region were missense mutations, the conversion of GTG codon to GCG codon in SNP3 resulted in the change of valine to alanine, thus p.Val17Ala, and the conversion of GAC codon to AAC codon in SNP5 resulted in the change of aspartic acid to asparagine, that is, p.Asp317Asn, and both SNPs were significantly associated with CVFD and MFC were significantly correlated, which may be due to the change in amino acid leading to the change in protein secondary structure, which in turn leads to the change in phenotype. SNP2, SNP4 and SNP6 were all located in the coding region and were synonymous mutations, but their variants are also significantly associated with wool traits, a result that is similar to the results of some previous studies [23,24,25]. Theoretically, synonymous mutations in coding regions usually do not cause alterations in the function of coded proteins, but the results of studies have shown that synonymous mutations are not completely silent, and some of them can affect the formation or loss of variable spliceosomes in genes, mRNA stability, and translation efficiency [26,27]. Some other synonymous mutations can affect protein folding and conformation, leading to alterations in gene function and phenotype [28]. It is important to note that the analysis of the association results between genotypes and wool traits showed that SNPs in non-coding regions (SNP1, SNP7 and SNP8) were also significantly associated with wool traits, which may be due to the fact that SNP in non-coding regions can influence the binding efficiency of transcription factors [29], affect mRNA shearing or alter DNA methylation modifications, which are involved in the process of gene expression regulation and phenotype generation, and that the SNP in the 3′UTR region of genes affects gene expression in multiple ways [30,31,32].

Haplotype analysis takes into account interactions between non-alleles and linkage disequilibrium between SNP, and is more comprehensive than analysis of individual SNPs [33]. In this study, two blocks were constructed and the results of the association analysis of the haplotype combinations haplotype combinations of the two blocks with wool traits showed that the haplotype combinations *H_2_H_3_* and *H_3_H_3_* of Block1 were significantly associated with wool MFC, and that MFC was significantly higher in the haplotype combinations *H_2_H_2_* and *H_2_H_3_* populations than in the *H_3_H_3_* population, which suggests that the presence of the haplotype *H2* in Block1 led to an increase in MFC; the haplotype combination *H_2_H_3_* of Block 2 was significantly associated with the MFD, CF and MFC, MFD was significantly higher in haplotype combination *H_1_H_3_* than in *H_2_H_3_*, while CF was significantly lower in diplotype *H_1_H_3_* than in *H_2_H_3_*. It should be noted that there is a correlation between fiber diameter and comfort factor, which is in agreement with the results of a previous study [34]. Since the *H_2_H_3_* in Block2 increases MFC while decreasing MFD, which can substantially improve wool quality, and its frequency of occurrence is high in the population, haplotype combination *H_2_H_3_* can be used as a selection target. The results of the association analyses of haplotype combinations with wool traits were generally consistent with the results of the single locus association analyses, both of which significantly affected wool traits. The effects of *KRT32* on wool traits suggest that this gene has a significant impact on the development of the hair follicle, and is an important gene in influencing wool phenotypes. It is important to note that we need to go further to understand the mechanism of the influence of other genes on wool traits as well as the interactions between the genes, to gain a deeper understanding of the formation of different types of wool, and better select and breed to meet human needs.

## 5. Conclusions

This study demonstrated for the first time the potential of the *KRT32* gene as a genetic marker for wool traits. *KRT32* was highly expressed in the skin and showed significant differences in expression at different stages of the skin, encoding proteins localized to the inner root sheath (IRS), outer root sheath (ORS) and dermal papilla (DP), and all eight SNPs in *KRT32* were significantly correlated with wool traits, including MFD, CF and MFC. Constructed haplotype after haplotype combination *H_2_H_3_* has higher CF and smaller MFD. The combination of the above indicates that the sequence variation of *KRT32* is significantly associated with economically important traits of wool (e.g., MFD and MFC), and it can be used as a candidate gene for wool improvement in Gansu Alpine Fine-wool sheep, which can provide a reference value for the improvement of wool quality.

## Figures and Tables

**Table 1:** Gene primers and internal reference sequence information.

Gene	GenBank Accession No.	Primer Sequence (5′-3′)	Product Size (bp)	Annealing Temperature (°C)
*KRT32*	XM_0040112899.5	F: GGACAGTGAGGACTGCAAGTT	185	60
		R: GCACACAAGGCACACAGACG		
*β-actin*	NM_001009784	F: AGCCTTCCTTCCTGGGCATGGA	113	60
		R: GGACAGCACCGTGTTGGCGTAGA		

**Table 2:** Primer information for the three exons.

Gene	Exon	Forward (5′→3′)	Reverse (5′→3′)	Product Length (bp)	Tm/°C
*KRT32* (XM_004012899.5)	1	TTAGCAGCTTCCTCTGGGATTGAGTC	GGGAAGTTTCTTTTCCCTGAATGTAGC	670	62
	6	TTTTAGTTGTTTGAGAAACTTCCACACTG	CACCATGAGTACCTGCCTGACTTCTC	468	60
	7	GAGCCGTTGGAAAGCACAAAGG	TTCGTCTGGAGCCCAACTGAGC	612	60

**Table 3:** PARMs-PCR primer sequences and fluorescent signal types.

SNP	Primer	Primer Sequences (5′–3′)	Fluorescent Signal	Genotype
g.21455859 T>C	Ft	GAAGGTGACCAAGTTCATGCTCTGCAGCCTTTCCGACGT	FAM	T
	Fc	GAAGGTCGGAGTCAACGGATTTGCAGCCTTTCCGACGC	HEX	C
	R	GACAGAGGAGGATCAGGGTCAG	/	/
g.21455953 G>A	Rc	GAAGGTGACCAAGTTCATGCTCAGGCTGGCAGATGTAACCC	FAM	G
	Rt	GAAGGTCGGAGTCAACGGATTCCAGGCTGGCAGATGTAACCT	HEX	A
	F	GACCCTGATCCTCCTCTGTCAG	/	/
g.21455976 T>C	Ft	GAAGGTGACCAAGTTCATGCTCATCTGCCAGCCTGGGGT	FAM	T
	Fc	GAAGGTCGGAGTCAACGGATTATCTGCCAGCCTGGGGC	HEX	C
	R	GCTAGTTGGGTGGTAGGTGGTG	/	/
g.21456106 G>A	Rc	GAAGGTGACCAAGTTCATGCTCAGCCAGGCGGCTGTTC	FAM	G
	Rt	GAAGGTCGGAGTCAACGGATTCAGCCAGGCGGCTGTTT	HEX	A
	F	GCAACGAGAAGGAGACCCTG	/	/
g.21460798 G>A	Fg	GAAGGTGACCAAGTTCATGCTGCAGGGCCTGGTCACCG	FAM	G
	Fa	GAAGGTCGGAGTCAACGGATTGCAGGGCCTGGTCACCA	HEX	A
	R	GGTCACAGCGGATCTCAGCC	/	/
g.21460884 A>G	Fg	GAAGGTGACCAAGTTCATGCTGCAGGGCCTGGTCACCG	FAM	A
	Fa	GAAGGTCGGAGTCAACGGATTGCAGGGCCTGGTCACCA	HEX	G
	R	GGTCACAGCGGATCTCAGCC	/	/
g.21463485 T>C	Ft	GAAGGTGACCAAGTTCATGCTCTGGTGCTTCCTGAGGCTGT	FAM	T
	Fc	GAAGGTCGGAGTCAACGGATTGGTGCTTCCTGAGGCTGC	HEX	C
	R	GCCCTGCTCTTTTGGTGG	/	/
g.21463503 C>G	Rg	GAAGGTGACCAAGTTCATGCTCTGCTCTTTTGGTGGCCG	FAM	C
	Rc	GAAGGTCGGAGTCAACGGATTCTGCTCTTTTGGTGGCCC	HEX	G
	F	ACTGGGTGGCTGGTGCTTC	/	/

**Table 4:** Distribution density of *K32* in various parts of skin tissues at various ages.

Names	Days	Corneum	Inner Root Sheath	Outer Root Sheath	Hair Medulla	Sebaceous Gland	Dermal Papilla
*KRT32*	1	+++	+++	+++	++++	++	+++
	30	+++	++++	++++	++++	++++	++++
	60	+++	++++	++++	++++	++++	++++
	90	+++	++++	++++	++++	++++	++++
	180	+++	++++	++++	++++	++++	++++
	270	+++	++++	++++	++++	++++	++++

Note: ++, Medium positive expression; +++, Strong positive expression; ++++, High positive expression.

**Table 5:** Genotype frequency and gene frequency of 8 SNPs of *KRT32*.

Gene	SNP	Locus	Polymorphism Information Content (PIC)	Genotype Frequency (n)			Gene Frequency	
*KRT32*	SNP1	g.21455859 T>C	0.3669	*TT* 0.223 (50)	*TC* 0.375 (84)	*CC* 0.402 (90)	T 0.410	C 0.589
	SNP2	g.21455953 G>A	0.3742	*GG* 0.231 (53)	*GA* 0.480 (110)	*AA* 0.288 (66)	G 0.472	A 0.528
	SNP3	g.21455976 T>C	0.3565	*TT* 0.140 (32)	*TC* 0.452 (103)	*CC* 0.408 (93)	T 0.366	C 0.634
	SNP4	g.21456106 G>A	0.3742	*GG* 0.231 (53)	*GA* 0.480 (110)	*AA* 0.288 (66)	G 0.472	A 0.528
	SNP5	g.21460798 G>A	0.3565	*GG* 0.140 (32)	*GA* 0.452 (103)	*AA* 0.408 (93)	G 0.366	A 0.634
	SNP6	g.21460884 A>G	0.3607	*AA* 0.153 (35)	*AG* 0.459 (105)	*GG* 0.389 (89)	A 0.382	G 0.618
	SNP7	g.21463485 T>C	0.2621	*TT* 0.712 (163)	*TC* 0.248 (59)	*CC* 0.031 (7)	T 0.840	C 0.159
	SNP8	g.21463503 C>G	0.1079	*CC* 0.887 (205)	*CG* 0.103 (24)	*GG* 0.008 (2)	C 0.939	G 0.061

**Table 6:** Association analysis of *KRT32* SNPs with wool traits.

SNPs	Genotype (n)	MFD (µm)	FDSD (µm)	CVFD	CF (%)	MSL (mm)	MSS (cN/dT)	MFC (^o^/mm)
SNP1	*TC* (84)	22.10 ± 2.86	5.69 ± 1.11	25.70 ± 3.17 ^a^	88.20 ± 11.37	72.81 ± 15.33	14.89 ± 6.33	106.44 ± 11.50 ^ab^
	*TT* (50)	21.79 ± 2.80	5.42 ± 0.90	24.85 ± 3.16 ^ab^	89.68 ± 10.29	71.78 ± 12.74	13.43 ± 5.74	109.48 ± 11.00 ^a^
	*CC* (90)	22.72 ± 3.00	5.60 ± 1.08	24.62 ± 3.16 ^b^	86.36 ± 12.64	73.28 ± 14.25	14.40 ± 5.95	102.98 ± 12.12 ^b^
SNP2	*GA* (110)	22.14 ± 2.90	5.62 ± 1.13	24.85 ± 3.35	88.23 ± 11.47	72.97 ± 15.07	14.57 ± 6.21	105.24 ± 11.52 ^ab^
	*GG* (53)	21.91 ± 2.80	5.47 ± 0.90	24.47 ± 2.42	89.25 ± 10.29	72.17 ± 12.60	13.67 ± 5.73	108.38 ± 11.12 ^a^
	*AA* (66)	22.79 ± 2.91	5.65 ± 1.01	24.44 ± 2.88	85.91 ± 12.85	73.52 ± 14.27	14.90 ± 5.66	102.48 ± 12.24 ^b^
SNP3	*TC* (103)	22.08 ± 2.87	5.65 ± 1.09	25.53 ± 3.05 ^a^	88.30 ± 11.34	72.86 ± 15.13	14.80 ± 6.30	107.26 ± 11.23 ^a^
	*TT* (32)	21.64 ± 2.65	5.38 ± 0.80	24.86 ± 2.43 ^ab^	90.41 ± 9.51	71.75 ± 12.22	13.17 ± 5.60	108.36 ± 11.89 ^a^
	*CC* (93)	22.75 ± 2.99	5.62 ± 1.07	24.67 ± 3.12 ^b^	86.22 ± 12.52	73.45 ± 14.11	14.57 ± 5.66	102.81 ± 12.00 ^b^
SNP4	*GA* (110)	22.188 ± 2.88	5.62 ± 1.33	25.29 ± 3.38	88.15 ± 11.42	72.70 ± 15.36	14.66 ± 6.22	105.83 ± 11.66 ^ab^
	*GG* (53)	21.91 ± 2.80	5.47 ± 0.89	24.92 ± 2.38	89.24 ± 10.29	72.17 ± 12.59	13.67 ± 5.73	108.82 ± 11.10 ^a^
	*AA* (66)	22.79 ± 2.99	5.65 ± 1.01	24.80 ± 2.87	85.90 ± 12.85	73.51 ± 14.32	14.90 ± 5.66	102.87 ± 12.22 ^b^
SNP5	*GA* (103)	22.08 ± 2.87	5.64 ± 1.09	25.53 ± 3.05 ^a^	88.30 ± 11.33	72.86 ± 15.14	14.79 ± 6.30	107.25 ± 11.23 ^a^
	*GG* (32)	21.64 ± 2.65	5.38 ± 0.80	24.86 ± 2.43 ^ab^	90.41 ± 9.51	71.75 ± 12.22	13.17 ± 5.60	108.36 ± 11.89 ^a^
	*AA* (93)	22.75 ± 2.90	5.62 ± 1.07	24.67 ± 3.13 ^b^	86.21 ± 12.52	73.45 ± 14.11	14.57 ± 5.66	102.80 ± 12.00 ^b^
SNP6	*AG* (35)	22.08 ± 2.95	5.62 ± 1.08	25.42 ± 3.06	88.21 ± 11.51	72.43 ± 15.22	14.69 ± 6.25	107.60 ± 11.17 ^a^
	*AA* (105)	21.66 ± 2.59	5.41 ± 0.81	24.96 ± 2.40	90.40 ± 9.39	72.31 ± 12.00	13.59 ± 5.98	107.91 ± 11.69 ^a^
	*GG* (89)	22.75 ± 2.94	5.64 ± 1.08	24.73 ± 3.15	86.28 ± 12.48	73.80 ± 14.03	14.53 ± 5.58	102.29 ± 11.93 ^b^
SNP7	*TC* (59)	22.38 ± 3.10	5.45 ± 0.91	24.36 ± 2.31 ^b^	87.46 ± 12.60	75.20 ± 14.59	15.09 ± 5.79	103.46 ± 12.20
	*TT* (163)	22.24 ± 2.83	5.65 ± 1.08	25.37 ± 3.21 ^a^	87.93 ± 11.21	72.21 ± 14.13	14.20 ± 6.04	106.20 ± 11.60
	*CC* (7)	22.24 ± 3.68	5.48 ± 1.40	24.43 ± 2.81 ^ab^	87.57 ± 15.22	71.00 ± 14.79	15.21 ± 5.06	109.30 ± 12.14
SNP8	*CG* (24)	23.175 ± 3.72 ^a^	5.59 ± 1.41	24.10 ± 2.37	83.67 ± 16.90 ^b^	75.83 ± 14.43	16.15 ± 6.48	102.00 ± 13.12
	*CC* (205)	22.20 ± 2.80 ^ab^	5.60 ± 1.04	25.20 ± 3.06	88.28 ± 10.83 ^a^	72.60 ± 14.25	14.26 ± 5.86	106.01 ± 11.59
	*GG* (2)	19.15 ± 0.64 ^b^	4.85 ± 0.07	25.35 ± 1.34	87.80 ± 11.65 ^ab^	72.00 ± 4.24	11.37 ± 3.63	105.58 ± 11.79

MFD, mean fiber diameter; FDSD, fiber diameter standard deviation; CVFD, coefficient of variation of fiber diameter; CF, comfort factor; MSL, mean staple length; MSS, mean staple strength; MFC, mean fiber curvature. ^a,b^ Values with different superscripts within the same column differ significantly at *p* < 0.05.

**Table 7:** Haplotypes and haplotype combinations of block1 and their respective frequencies.

Haplotype	SNP2	SNP3	SNP4	SNP5	Frequency/%	Haplotype Combination	Frequency/%	Haplotype Combination	Frequency/%
*H_1_*	A	C	A	A	52.8	*H_1_H_1_*	28.9	*H_2_H_2_*	14.0
*H_2_*	G	T	G	G	36.6	*H_1_H_2_*	37.2	*H_2_H_3_*	7.9
*H_3_*	G	C	G	A	6.1	*H_1_H_3_*	10.5	*H_3_H_3_*	1.4
*H_4_*	G	T	G	A	4.5				

**Table 8:** Association analysis of wool traits with haplotype combinations of Block1.

Genes	Haplotype Combination	MFD (µm)	FDSD (µm)	CVFD	CF (%)	MSL (mm)	MSS (cN/dT)	MFC (^o^/mm)
*KRT32*	*H_1_H_1_*	22.79 ± 0.37	5.65 ± 0.13	24.80 ± 0.35	85.91 ± 1.58	73.51 ± 1.76	14.90 ± 0.69	102.87 ± 1.50 ^ab^
	*H_1_H_2_*	22.08 ± 0.31	5.68 ± 0.12	25.67±±0.34	88.28 ± 1.23	73.08 ± 1.68	14.98 ± 0.69	106.36 ± 1.24 ^ab^
	*H_1_H_3_*	22.50 ± 0.64	5.45 ± 0.26	24.11 ± 0.79	87.58 ± 2.49	72.63 ± 2.93	13.28 ± 1.16	103.27 ± 2.46 ^ab^
	*H_2_H_2_*	21.64 ± 0.47	5.38 ± 0.14	24.86 ± 0.43	90.41 ± 1.68	71.75 ± 2.16	13.16 ± 0.99	108.36 ± 2.10 ^a^
	*H_2_H_3_*	22.07 ± 0.73	5.50 ± 0.25	24.83 ± 0.57	88.39 ± 2.76	71.83 ± 3.29	13.90 ± 1.44	111.48 ± 2.17 ^a^
	*H_3_H_3_*	23.90 ± 1.45	6.23 ± 0.29	26.20 ± 1.06	82.00 ± 5.03	73.67 ± 2.27	17.67 ± 2.65	97.70 ± 3.74 ^b^
	*p* value	0.094	0.0880	0.136	0.122	0.309	0.103	0.029

MFD, mean fiber diameter; FDSD, fiber diameter standard deviation; CVFD, coefficient of variation of fiber diameter; CF, comfort factor; MSL, mean staple length; MSS, mean staple strength, MFC, mean fiber curvature. ^a,b^ Values with different superscripts within the same column differ significantly at *p* < 0.05.

**Table 9:** Haplotypes and haplotype combinations of block2 and their respective frequencies.

Haplotype	SNP6	SNP7	Frequency/%	Haplotype Combination	Frequency/%	Haplotype Combination	Frequency/%
*H_1_*	G	T	46.4	*H_1_H_1_*	20.0	*H_2_H_2_*	15.3
*H_2_*	A	T	38.1	*H_1_H_2_*	35.8	*H_2_H_3_*	10.0
*H_3_*	G	C	15.5	*H_1_H_3_*	15.7	*H_3_H_3_*	3.1

**Table 10:** Association analysis of wool traits with haplotype combinations of Block2.

Genes	Haplotype Combination	MFD (µm)	FDSD (µm)	CVFD	CF (%)	MSL (mm)	MSS (cN/dT)	MFC (^o^/mm)
*KRT32*	*H_1_H_1_*	22.48 ± 0.38 ^ab^	5.69 ± 1.17	25.27 ± 0.54	87.63 ± 1.58 ^ab^	73.46 ± 2.01	13.68 ± 0.81	103.21 ± 1.66 ^ab^
	*H_1_H_2_*	22.36 ± 0.34 ^ab^	5.73 ± 0.13	25.61 ± 0.36	87.04 ± 1.34 ^ab^	71.46 ± 1.69	14.75 ± 0.70	107.14 ± 1.28 ^a^
	*H_1_H_3_*	23.19 ± 0.55 ^a^	5.59 ± 0.16	24.10 ± 0.40	84.31 ± 2.34 ^b^	74.78 ± 2.45	15.48 ± 0.96	99.76 ± 2.06 ^b^
	*H_2_H_2_*	21.66 ± 0.44 ^ab^	5.41 ± 0.14	24.96 ± 0.41	90.40 ± 1.59 ^ab^	72.31 ± 2.03	13.59 ± 1.01	107.91 ± 1.98 ^a^
	*H_2_H_3_*	21.10 ± 0.49 ^b^	5.23 ± 0.16	24.77 ± 0.45	92.39 ± 1.66 ^a^	75.87 ± 3.07	14.48±±1.24	109.25 ± 1.99 ^a^
	*H_3_H_3_*	22.24 ± 1.39 ^ab^	5.48 ± 0.53	24.43 ± 1.06	87.57 ± 2.75 ^ab^	71.00 ± 5.59	15.21 ± 1.91	109.30 ± 0.78 ^a^
	*p* value	0.026	0.174	0.153	0.027	0.339	0.373	0.005

MFD, mean fiber diameter; FDSD, fiber diameter standard deviation; CVFD, coefficient of variation of fiber diameter; CF, comfort factor; MSL, mean staple length; MSS, mean staple strength, MFC, mean fiber curvature. ^a,b^ Values with different superscripts within the same column differ significantly at *p* < 0.05.

## Data Availability

The authors confirm that the graphs in the article contain all the data needed to substantiate the article’s conclusions.
